# Determination of the effective swath of a plant protection UAV adapted to mist nozzles in mountain Nangguo pear orchards

**DOI:** 10.3389/fpls.2024.1336580

**Published:** 2024-06-21

**Authors:** Yihan Liu, Weixiang Yao, Shuang Guo, Hao Yan, Ziqi Yu, Sikai Meng, Dennis Chen, Chunling Chen

**Affiliations:** ^1^ College of Information and Electrical Engineering, Shenyang Agricultural University, Shenyang, China; ^2^ Eavision Technologies Co., Ltd, Jiangsu, China; ^3^ Liaoning Engineering Research Center for Information Technology in Agriculture, Shenyang, Liaoning, China

**Keywords:** effective swath, flight parameters, mist nozzle, mountain orchards, UAV/UAS

## Abstract

Plant protection unmanned aerial vehicles (UAVs) have become popular in mountain orchards, but due to the differences in planting structures, the chances of heavy spraying, missed spraying and pesticide drift are increasing. To mitigate the adverse effects of these phenomena, it is necessary to clarify the effective deposition range of aerial spray droplets. This study proposed an effective spray swath determination method for the effective spraying range of mountainous orchards with UAVs equipped with a mist nozzle (bilateral 1% coverage). This approach focused on exploring the effects of flight height (unidirectional flight modes of 2, 3 and 4 m), spray nozzle atomization performance (reciprocating flight modes of 20, 30 and 40 µm) and flight route (treetop flying and inter-row flying) on the spraying range in a mountain setting. In addition, the study analysed the relationship between the droplet-size spectrum and the effective swath position. The results showed that it is feasible to use the bilateral 1% coverage evaluation method to determine the effective spray swath of a UAV adapted with a mist nozzle for aerial operation in a mountainous Nangguo Pear orchard. With the increase in UAV flight height (2–4 m), the effective unidirectional spray swath also increased, and with the increase in atomization level (20–40 μm), the effective reciprocating spray swath showed a decreasing trend. Moreover, the average effective swath width measured by the UAV for treetop flight was greater than that measured for inter-row flight. The study also found that the proportion of small droplets (droplet size less than 100 µm) below the UAV route was lower (approximately 50%) than along the sides of the route (approximately 80%), and the spray swath was not symmetrically distributed along the flight route but shifted laterally by approximately 3 to 4 m in the downhill direction.

## 1 Introduction

As a new type of plant protection, plant protection unmanned aerial vehicles (UAVs) are also known as Unmanned Arial Systems (UAS), plant protection UAV aerial application has the advantages of high spraying efficiency, good atomization, and suitability for a variety of terrains (Xavier et al., 2019; Jiang et al., 2022; Sinha et al., 2022), and has been widely used in recent years. However, due to various factors, such as operating parameters and airflow, the aerial application of pesticides by plant protection UAVs has risks, such as low pesticide utilization and droplet drift, resulting in serious pesticide residues or loss, polluting the environment and endangering public safety, so there is a high demand for accuracy in the application process (Chen et al., 2020; Itmec et al., 2022).

The effective swath is an important metric for evaluating the accuracy of aerial application by plant protection UAVs, i.e., the width of the coverage area where droplets are effectively deposited during aerial spraying operations by plant protection UAVs is an important reference for determining spraying operation parameters and an important reference indicator for UAV application path planning (Richardson et al., 2019). When plant protection UAVs are used for spraying operations, setting the effective swath width requires careful balancing of different considerations. To maximize application coverage, leverage the convenience of UAVs and ensure operational efficiency, the swath width cannot be set too small. It is also important to maximize the quality of the operation, increase the effective deposition of droplets in the target area and reduce drift, so the swath width should not be set too wide (Chen et al., 2017). If the changing nature of the swath width is ignored during flight, respraying or misspraying may occur, resulting in wasted plant protection products and environmental pollution. Accurate and effective determination of the swath width is a useful tool for ensuring operational efficiency, improving the quality of spraying operations, reducing drift, and ensuring environmental protection.

Notably, plant protection UAVs have been widely used on field crops (rice, wheat, soybeans, cotton) in recent years, and the related research results have been informative (Lou et al., 2018; Qin et al., 2018; Chen et al., 2021; Zhou et al., 2024). Researchers have also carried out studies on flatland settings where field crops are commonly planted. Currently, fruit tree applications in mountain settings have become a topic of interest for scholars in the field, and researchers have carried out research around typical spraying operation parameters such as flight height (Hou et al., 2019; Souza et al., 2022), flight mode (Biglia et al., 2022), meteorological conditions (Wang J. et al., 2018), UAV models (Wang et al., 2021; Ran et al., 2022), tree shapes (Zhang et al., 2016), nozzle parameters (Sarri et al., 2019; Chen et al., 2022) and fruit tree canopy positions (Zhang et al., 2017; Tang et al., 2018). Relevant studies have also made progress in the deposition and drift characteristics of fruit trees (Wang G. B. et al., 2020; Guo et al., 2021; Wang et al., 2023), but few studies have reported the effects of spray swaths on spray application. Especially in mountain orchards, the conventional means of exploring crop deposition and drift characteristics usually involve setting fixed swath width values (Giles and Billing, 2015; Wang C. L. et al., 2020; Yao et al., 2022). However, the swath width is influenced by the way the plant protection UAVs operate in the air and is not necessarily constant. This can change depending on many factors, such as flight mode, flight height, and operating scene. Setting a fixed swath width ignores the effect of swath width variation on the effectiveness of the UAV’s application effect and thus also affects the accurate determination of droplet deposition and drift characteristics.

Nozzle atomization performance is also a key factor in the effectiveness of plant protection UAV applications, but it should be noted that nozzle atomization performance also affects the effective swath. Although the small size of the nozzle droplets allows for greater droplet coverage, drift may occur, and the probability of the spray mixture settling on nontarget crops increases, thus polluting the environment. A larger droplet size results in fewer droplets, less effective coverage, and a smaller separation between operating routes, resulting in less efficient UAV operation (Chen et al., 2020). Therefore, choosing the right aerial nozzle for plant protection UAV operation is vital. Currently, most of the conventional aerial nozzles used by plant protection UAVs have droplet sizes in the range of 50 to 350 µm (Brown and Giles, 2018; Wang X. N. et al., 2018; Hussain et al., 2022). Compared to conventional pressure and centrifugal spray nozzles, mist nozzles operate with finer droplet size atomization and have greater penetration, gradually showing better operational results in mountain orchard pest control. Eavision Technologies Co., Ltd. developed and launched a double-peaked mist nozzle as an example. This nozzle has a waterproof construction and can be continuously adjusted from 20 µm to 250 µm droplet sizes. At the same time, the nozzle produces droplets with a “dual-peak droplet size” characteristic; that is, there are two droplet sizes, resulting in large droplets of good orientation that are less likely to drift. The small droplets of mist produced penetrate the dense canopy of fruit trees. At the same time, the combination of the nozzle with its wind device can achieve “wind and mist synchronization”, reducing the impact of ambient wind speed and rotor airflow on the actual operation. To a certain extent, it can also enhance the application amount on the leaf abaxial surface, so it is gradually applied in mountain orchards. However, the combination of mist nozzles and plant protection UAVs is rare. At present, there are also relatively few studies on the spray swath characteristics of mist nozzles and a lack of spray swath data for collaborative operations with plant protection UAVs, and the actual operational effects have yet to be clarified.

Researchers have already conducted studies on the effective swath of aerial spraying using a variety of determination methods. Zhang et al. (2015) evaluated three different determination methods (greater than the maximum range of the mean percentage coverage, a range of CV less than 20%, and a deposition rate on either side of the flight centerline greater than half the height of the peak one-way distribution as the basis for determining the effective swath) to accurately determine the effective swath for two types of fixed-wing aircraft. Their results showed that the measured swath width was more stable when the deposition rate on either side of the flight centerline was greater than half the peak height of the distance of the one-way distribution as the effective swath determination method. They used a manned fixed-wing aircraft, and the spraying equipment was different from that of plant protection UAVs, so this study can be used as a reference for determining the effective swath of plant protection UAVs. Martin and Latheef (2022) focused on the effect of payload, type, flight height, and operating speed on the effective swath based on different models of pressure nozzles. They reported that the average effective swath widths were 5.60, 8.74, 10.11, and 10.60 m with increasing payloads (5, 10, 15, and 20 L), respectively, with an increasing trend in the effective swath widths. At the same time, the flight height and flight speed of different UAV models had different effects on the effective swath. This view was similarly confirmed in another report by Martin et al. (2019). Yao et al. (2021) proposed a new method for determining the effective swath based on helicopter application: the furthest sampling site on either side of the route where the deposition reached 1.000 µL/cm^2^ was defined as the starting and ending points of the effective swath area, and the distance between these two points was considered the effective swath width. Based on this method, the effect of 3 different types of CP nozzles on the effective spray swath width of the helicopter was studied, and the results showed that the nozzle types have significant effects on the spray swath width. There was another report by Yao et al. (2019) on the effective swath of helicopters. They found that as the flight speed increased, the effective swath width first increased slowly but then decreased sharply. The above studies showed that most of the existing reports on effective swaths have been carried out on manned aircraft or UAVs in flatland settings, and most have been adapted to conventional hydraulic nozzles for research. Few studies have been carried out on the variation in the effective swath of plant protection UAVs based in mountain orchards.

In summary, there is an urgent need for research into the evaluation and factors influencing the effective swath of plant protection UAVs adapted to mist nozzles, especially for mountain orchards with a certain spacing between planting rows, a dense canopy structure, and a complex and special topography. The use of plant protection UAVs for applying pesticides has raised environmental and regulatory concerns. Guo et al. (2022) evaluated the droplet deposition characteristics of four kinds of spray nozzles with different atomization performances in a Nangguo Pear mountain orchard setting, but the study used a tree-top hovering operation and only analysed the effect of spray nozzle atomization performance on the droplet deposition characteristics without considering the effect of the variation in spray swath. Based on this research, the team decided to focus on effective swath determination research for mountain orchard scenarios. The main purpose of this study is to propose a new effective spray swath determination method for plant protection UAVs in mountain Nangguo pear orchards adapted to mist nozzles. At the same time, the study also focuses on the influence of factors such as flight mode, flight height, and spray nozzle atomization performance on the effective spraying range of mountain Nangguo pear orchards. The results of the study can promote the development of plant protection UAV technology, which will also have a positive effect on the ecological protection of mountain Nangguo pear orchards.

## 2 Materials and methods

The test was carried out in two sessions: a unidirectional effective swath width measurement test (USW) and a reciprocating effective swath width measurement test (RSW). The test subjects were all Nanguo pear trees. The USW site was in Shanxi Gou village, Haicheng city, Liaoning Province, China (40°51'55"N, 122°48'42"E), on 17 July 2022. The RSW was based on the analysis of the results of the USW and was optimized. The test site was in Wangjia Village, Jiwen Town, Haicheng City, Liaoning Province, China (40°46'9"N, 123°3'6"E), and the test was conducted on August 1, 2022.

### 2.1 UAV spraying systems

**Figure 1:**
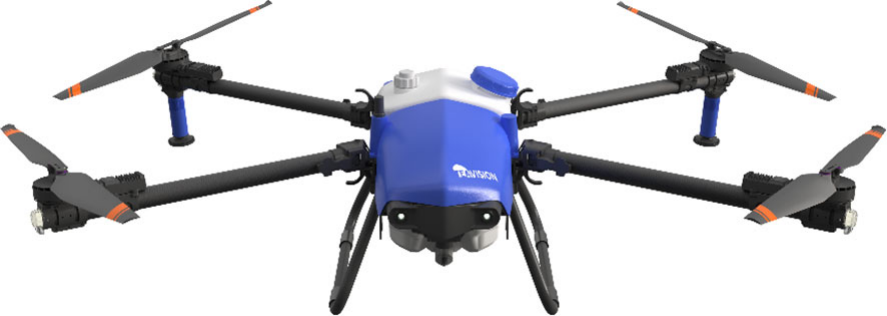
The EA-30X UAV.

An EA-30X UAV (Eavision Technologies Co., Ltd., Jiangsu, China) was used for the aerial spraying tests in this study, as shown in **Figure 1**. The EA-30X UAV was equipped with binocular environmental sensing technology and a CCMS-L20000 double-peaked mist nozzle. The nozzle atomization level can be divided into five levels: 10 µm, 20 µm, 30 µm, 40 µm and 100 µm. Notably, this atomization level was proposed by Eavision Technologies Co., Ltd., Jiangsu. The relevant atomization particle size in this paper only represents a qualitative description of the atomization performance. The actual droplet size is not fixed to this size but fluctuates within a certain range, and the study in this paper has also carried out relevant measurements. At the same time, the nozzle atomization is also characterized by a dual-peak droplet size, which enhances the directionality of large droplets as well as the penetration coefficient of small droplets, helping to penetrate various thick canopy vegetation. In addition, the onboard ultrasonic flow meter was measured to have a flow error of less than 5%, meeting the conditions for accurate spraying. The main performance of this plant protection UAV is shown in **Table 1**.

**Table 1:** Performance parameters of the EA-30X UAV.

Main parameters	Specifications and values
Effective swath/m Nozzle number Types of nozzles Atomization droplet size range/µm Pesticide tank capacity/L Number of rotors Boom spread dimensions (length × width × height)/(mm × mm × mm) Diaphragm pump flow/(L/min)	7–8 2 CCMS-L20000 double-peaked mist nozzle 20–250 30 4 2350× 2760 × 620 0.5–10

The effective swath width and nozzle droplet size range are the range of the parameters provided by Eavision Technologies Co., Ltd. for operation on flatland scenes. The reference flight height for the UAV parameters is 2–5 m.

### 2.2 Test treatments and sampling site settings

To obtain a suitable method for determining the effective swath and to accurately determine how the effective swath width varied with flight mode, flight height, and nozzle atomization performance, the test was set up with 18 treatments in USW (T1–T9) and RSW (T10–T18). **Table 2** shows the parameters for each trial.

**Table 2:** Operational parameter settings for USW and RSW with the UAV.

Mode of operation	Test treatment	Spray volume /(L/ha)	Flight speed /(m/s)	Assignment scene	Flight mode	Flight height/m	Nozzle atomization performance/µm
Unidirectional	T1	60	2.0	Flatland scene		2.0	30
	T2					3.0	30
	T3					4.0	30
	T4			Mountain scene	Flying along the treetops	2.0	30
	T5					3.0	30
	T6					4.0	30
	T7				Flying along the inter-row	2.0	30
	T8					3.0	30
	T9					4.0	30
Reciprocating	T10			Flatland scene		3.0	20
	T11					3.0	30
	T12					3.0	40
	T13			Mountain scene	Flying along the treetops	3.0	20
	T14					3.0	30
	T15					3.0	40
	T16				Flying along the inter-row	3.0	20
	T17					3.0	30
	T18					3.0	40

Flight height is the distance of the UAV from the top of the canopy.

**Figure 2:**
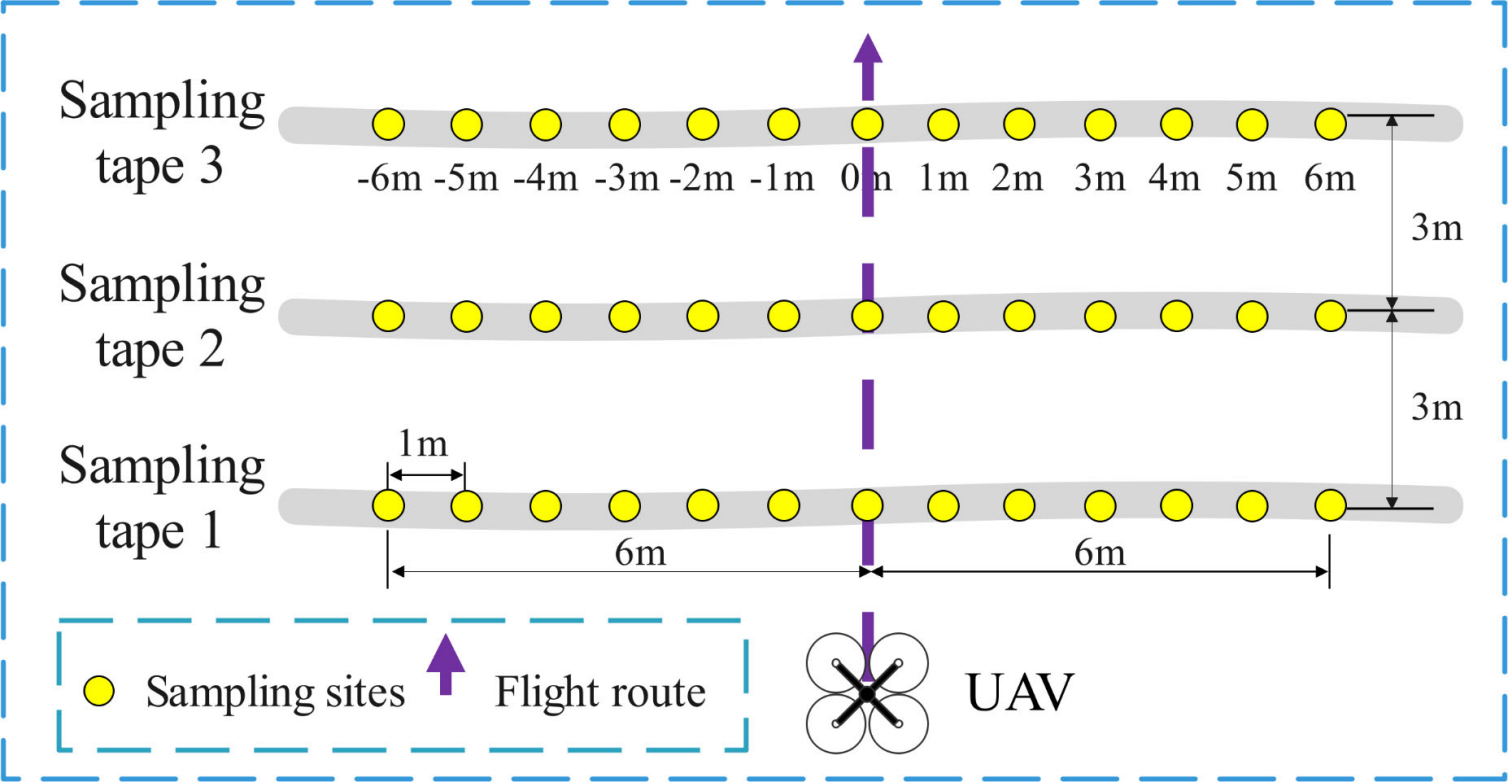
The flatland scene layout and flight route of the USW. Each sampling tape was marked by 1 m interval from -6 m to 6 m, and 0 m was the route position.

Both the USW and RSW included a flatland scene and a mountain scene. To carry out repeated tests, as well as to protect the environment and reduce pollution, water was used as the spraying reagent for all tests. As shown in **Figure 2**, three droplet sampling tapes perpendicular to the direction of the UAV flight path were set up for the USW in the flatland scene, adjacent sampling tapes were spaced 3 m apart, and 13 sampling sites were set at 1 m intervals for each sampling tape. The study used a droplet collection card-water sensitive paper (WSP, 26 × 76 mm; Syngenta Crop Protection AG, Basel, Switzerland) to collect key information such as the coverage, droplet size, and density of droplet deposition. The USW in the flatland scene consisted of three treatments set at different flight heights of 2 m (T1), 3 m (T2), and 4 m (T3). At the same time, for reference to the actual operation situation, the constant setting of the UAV flight speed is 2 m/s, the spraying volume is 60 L/ha, and the flight mode is along the treetops.

**Figure 3:**
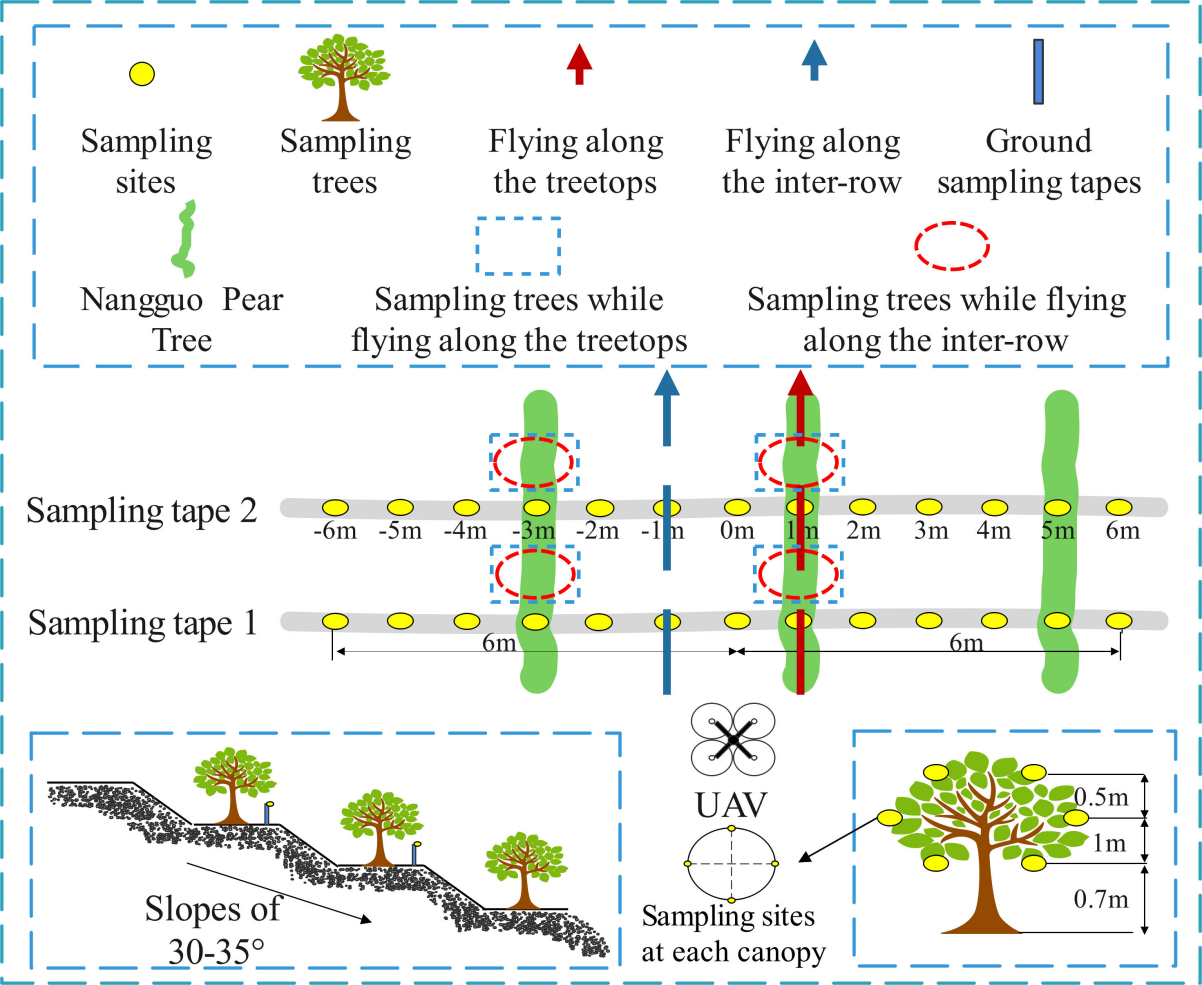
The mountain scene layout and flight route of the USW. Each sampling tape was marked 1 m apart from -6 m to 6 m, when flying along the treetops, the route position was at 1m, and when flying along the inter-row, it was at -1m. The trees are equally spaced in the diagram.

As shown in **Figure 3**, the USW in the mountain scene had a mountain slope of 30 to 35°, and the fruit trees were arranged transverse to the slope lines. The average plant spacing and average row spacings were 4.95 m and 5.1 m, respectively, and the average tree height was 2.4 m. The planting density was 396 plants/ha, and the canopy dimensions of the canopy sampling area were 2.4 × 4.3 × 4.15 m (height × diameter along the row × diameter across the row). The upper, middle, and lower canopy levels were 0.7 m, 1.7 m and 2.1 m from the ground, respectively. The test was carried out in six treatments (T4–T9), and the flight height and other operational parameters were set at the same level as in the flatland scene to enable the comparison of the effective swath width of the flatland and mountain in the analysis. According to the characteristics of the fruit trees, for two types of flight, treetop flying (T4–T6) and inter-row flying (T7–T9), the flight height setting for each treatment was the same as for the flatland scene. Two identical sampling areas were set up, each consisting of the tree canopy sampling area and the ground sampling area. The canopy sampling area was on both sides of the tree canopy perpendicular to the direction of flight; 4 pieces of water-sensitive paper were arranged in 4 directions of the upper, middle and lower layers of the canopy (the vertical direction of the tree), and a total of 12 pieces were arranged at the three layers. For each processing, two characteristic fruit trees above each ground collection area were selected, totalling 4 trees. With reference to the spatial position sampling at the bottom of the canopy, the ground sampling area was set below the sampling area of the canopy, at the same height as the lower part of the tree crown, and the height was approximately 0.7 m from the ground. Because it is necessary to ensure that droplets can penetrate the tree canopy, as long as the deposition effect of the ground sampling area meets the evaluation conditions, it is proven that the tree canopy also has a good deposition effect. The ground collection area consists of two droplet sampling tapes with an interval of 5 m, and 13 sampling sites are arranged at an interval of 1 m for each sampling tape.

**Figure 4:**
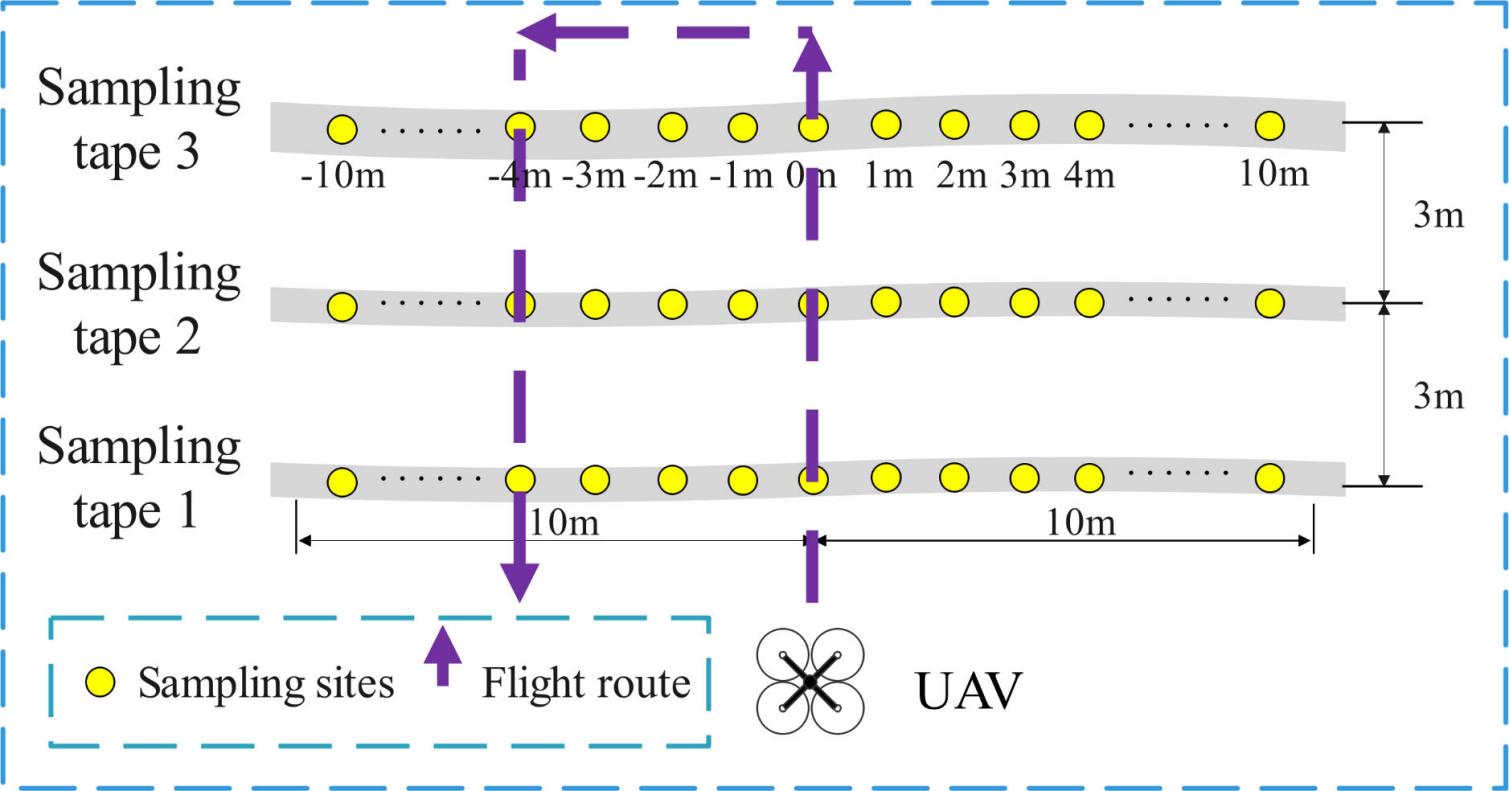
The flatland scene layout and flight route of the RSW. Each sampling tape was marked by 1 m interval from -10 m to 10 m, and the UAV flew along the route position of 0 m and returned at the route position of -4 m.

The setting of operational parameters for the RSW was based on the actual spraying effect of the USW, and the RSW also set up a flatland scene and a mountain scene. As shown in **Figure 4**, the operating speed and spray volume of the flatland scene were the same as in the USW, at 2 m/s and 60 L/ha, respectively, and the flying mode was treetop flying. The droplet size levels of different nozzles were set for 3 treatments in the test, which were 20 µm, 30 µm and 40 µm. In addition, the USW researches the effect of flight height change on the effective swath, and the RSW researches the effect of nozzle atomization performance on the effective swath. Meanwhile, according to the relevant parameters given by Eavision Technologies Co., Ltd. and the empirical height of 3 m summarized from the daily operation, the results of the USW also prove that the operation at 3 m is better. Therefore, the flight height for the RSW was set at 3 m.

**Figure 5:**
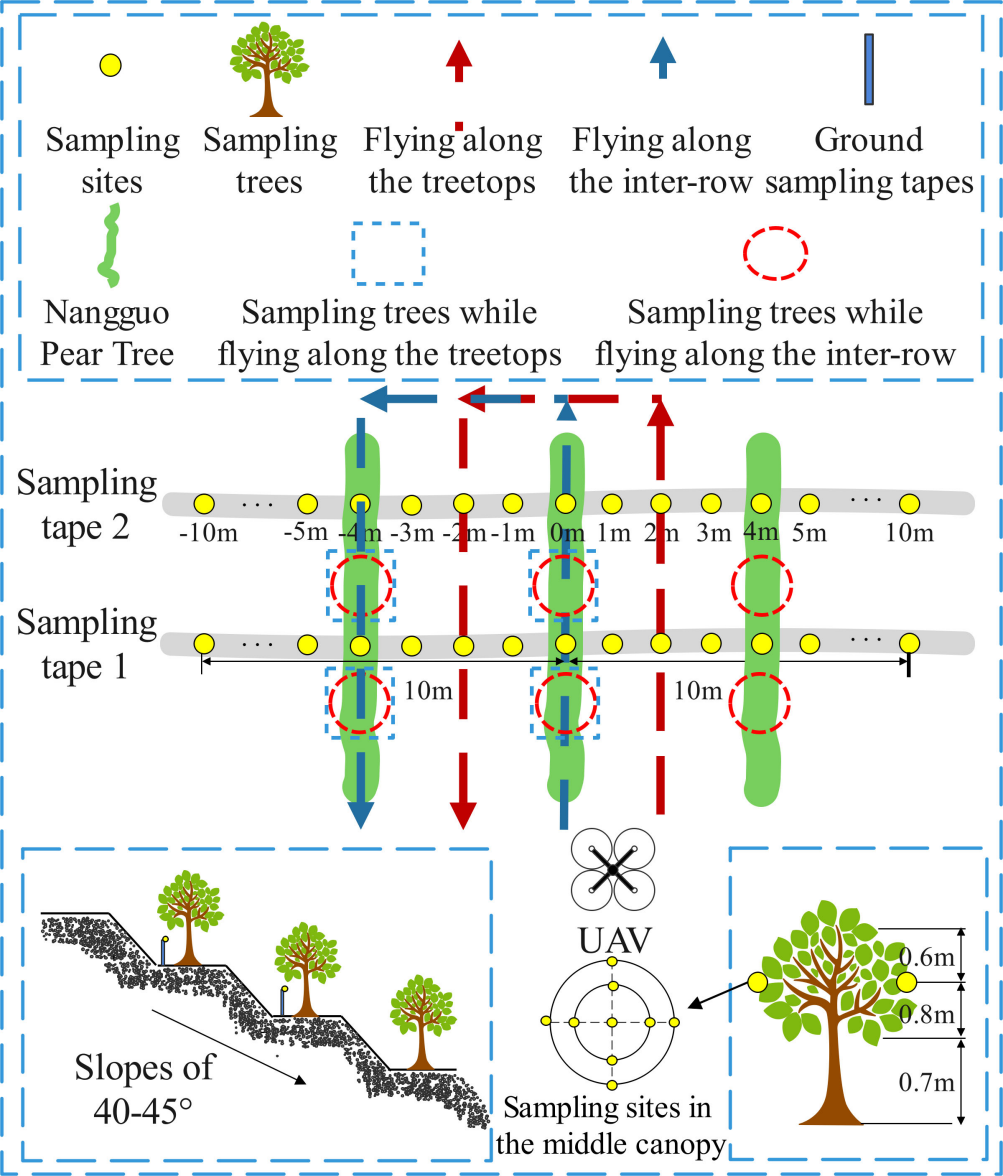
The mountain scene layout and flight route of the RSW. Each sampling tape was marked with a 1 m interval from -10 m to 10 m. When flying along the treetops, the UAV flew along the route position of 0 m and returned at the route position of -4 m, and when flying along the inter-row, it was at -1 m, the UAV flew along the route position of 2 m and returned at the route position of -2 m. The trees are equally spaced in the diagram.

As shown in **Figure 5**, the mountain scene of the RSW was near the flatland scene of the RSW, the slope of the hill was 40 to 45°, the fruit trees were arranged transverse to the slope lines, the average spacing between plants and rows was 3.5 m and 5.8 m, respectively, the average tree height was 2.4 m, the planting density was 492 plants/ha^-1^, the canopy size of the canopy sampling area was 2.4 × 4.3 × 4.15 m (height × diameter along the row × diameter across the row), and the upper, middle and lower layers of the canopy were 0.7 m, 1.5 m and 2.1 m above the ground, respectively. The operating parameters for the RSW in the mountain scene were set according to the flatland scene operating mode, corresponding to setting the nozzle droplet size level, and the flight mode was set to enable treetop flying (T13–T15) and inter-row flying (T16–T18). Each flight mode corresponded to the inclusion of 3 treatments with the same nozzle atomization performance settings as the flatland scene. The test also had two identical sampling areas, each consisting of the tree canopy sampling area and the ground sampling area. The canopy sampling area was in the middle of the canopy (different sampling sites on each tree were located at the same level), with nine water-sensitive papers arranged in an inner and outer ring. With the UAV flying along the treetops, two characteristic fruit trees above each ground sampling tape were selected for each treatment, for a total of four trees. Under the method of UAV inter-row flying, three characteristic fruit trees were selected above each ground sampling area, for a total of six trees. As with the USW setup, the ground sampling area was located below the canopy sampling area; again, two droplet sampling tapes were included, spaced 3.5 m apart, and the sampling location was at the same height as the lower part of the canopy, approximately 0.7 m. Each sampling tape was spaced at 1 m intervals with 21 sampling sites.

### 2.3 Monitoring of meteorological conditions

Weather conditions, including temperature, relative humidity, wind speed, and direction, were recorded in real time using a Kestrel 5500 Link micro weather station (Nielsen-Kellerman, Minneapolis, Minnesota, USA) during the test. The weather station was set up high away from the test area, and the recording interval was 5 s. Due to the large number of tasks set up in field trials, the long time spent on single treatment, and the complex and changeable field weather, it is impossible to ensure that the meteorological conditions of each treatment are exactly the same, and the experiment can only be carried out on the premise of relatively close weather conditions. On the whole, the meteorological conditions were relatively stable during the test; the USW measured average temperatures of 24.3°C and 24.6°C in the flatland scene and mountain scene, the average humidity was 83.9% and 81.4%, respectively, and the wind speed remained between 0.83 and 1.69 m/s and 0 and 0.38 m/s, respectively. The average temperatures measured by RSW in the flatland and mountain scenes were 29.6°C and 31.1°C, respectively, and the average humidities were 82.6% and 71.4%, respectively, with wind speeds between 0 and 0.5 m/s and 0.18 and 0.92 m/s, respectively. The meteorological data values for each treatment are shown in **Table 3**.

**Table 3:** Meteorological conditions.

Mode of operation	Assignment scene	Test treatment	Temperature/°C	Relative humidity/%	Wind speed/(m/s)
Unidirectional	Flatland scene	T1	23.91 ± 0.11	84.83 ± 0.56	1.69 ± 0.69
		T2	24.71 ± 0.31	83.18 ± 0.77	0.83 ± 0.52
		T3	24.38 ± 0.12	83.82 ± 0.47	1.45 ± 0.46
	Mountain scene	T4	25.97 ± 0.17	77.71 ± 0.74	0.14 ± 0.19
		T5	25.43 ± 0.42	83.02 ± 0.44	0.26 ± 0.41
		T6	24.92 ± 0.13	85.03 ± 0.21	0.38 ± 0.39
		T7	25.75 ± 0.08	81.98 ± 0.31	0.06 ± 0.14
		T8	25.74 ± 0.20	80.48 ± 0.31	0.18 ± 0.35
		T9	20.06 ± 0.05	80.45 ± 0.27	0
Reciprocating	Flatland scene	T10	29.93 ± 0.08	83.2 ± 0.84	0
		T11	29.56 ± 0.05	81.13 ± 0.12	0.5 ± 0.87
		T12	29.21 ± 0.03	83.52 ± 0.22	0.28 ± 0.45
	Mountain scene	T13	32.42 ± 0.32	67.69 ± 0.63	0.18 ± 0.29
		T14	30.93 ± 0.13	70.27 ± 0.17	0.92 ± 0.23
		T15	32.05 ± 0.21	66.62 ± 0.51	0.19 ± 0.26
		T16	30.88 ± 0.13	71 ± 0.57	0.78 ± 0.18
		T17	30.52 ± 0.23	73.78 ± 0.52	0.61 ± 0.35
		T18	29.64 ± 0.12	79.18 ± 0.39	0.52 ± 0.46

The numerical data in the table are means ± standard deviations.

### 2.4 Data collection and analysis

At the end of each field test, the laid-out water-sensitive papers were sequentially numbered, taken back to the laboratory, and scanned at 600 dpi resolution using an EPSON DS-1610 scanner (Epson (China) Co., Ltd., Beijing, China). The scanned data were processed for image analysis using DepositScan software (United States Department of Agriculture, Wooster, USA), and evaluation indicators such as Dv_0.1_, Dv_0.5_, and Dv_0.9,_ as well as the coverage of the droplet and droplet deposition, were obtained. At the same time, referring to the study of Guo et al. (2022), they experimented with the pressure nozzles with large droplet sizes, which were categorized into five intervals of ≤100 µm, 101–200 µm, 201–300 µm, 301–400 µm, and 401- 500 µm. Since this study used mist nozzles with a small droplet size, preliminary statistics on the droplets revealed that the droplet size range was mainly within 200 µm. Therefore, for the accuracy of data analysis, the droplet size spectrum was divided into four intervals of<100 µm, 100–150 µm, 150–200 µm and >200 µm. In addition, information about the penetration coefficient and the distribution span of droplet size was calculated. The permeability coefficient in the vertical direction is denoted by Pv, and the permeability coefficient in the horizontal direction is denoted by Ph (Wang et al., 2022). The calculation formula is shown in Equations 1, 2:


(1) $${\text{P}}_{\text{v}}={\text{C}}_{\text{low}}/{\text{C}}_{\text{up}\&\text{mid}}\times 100\%$$

(2) $${\text{P}}_{\text{h}}={\text{C}}_{\text{i}}/{\text{C}}_{\text{e}}\times 100\%$$

where C_low_ is the coverage (%) in the lower part of the tree canopy, C_up&mid_ is the sum of the coverage (%) of the upper and middle layers of the tree canopy, C_i_ is the coverage (%) of the inner ring of the canopy sampling, and C_e_ is the coverage (%) of the outer ring of the canopy picking.

In addition, in this study, it used the relative droplet-size spectral width (RS) to evaluate the droplet size distribution characteristics, also known as the distribution span of droplet size. The smaller the RS is, the more concentrated the droplet size distribution. When RS = 1, the droplet size is symmetrically distributed. The formula is shown in Equation 3:


(3) $$\text{RS }=\left({\text{Dv}}_{0.9}-{\text{Dv}}_{0.1}\right)/{\text{Dv}}_{0.5}$$

In the formula, D_V0.1_, D_V0.5_, and D_V0.9_ indicate the droplet size from small to large accumulating to 10%, 50%, and 90% of the total droplet volume, respectively, corresponding to the droplet size values.

### 2.5 Method for determining effective swath

For the determination of the effective swath, two conventional methods were used in this study: an evaluation method of droplet density and an evaluation method of 50% effective deposition. After consultation, some scholars (Li et al., 2022; Wang et al., 2022; Qi et al., 2023) have used droplet coverage of more than 1% as an indicator of effective droplet deposition. Based on the research of relevant scholars on the droplet deposition effect and the actual operation effect under a mist nozzle, a new method for determining the effective swath was proposed: the bilateral 1% coverage evaluation method—a comparative analysis of these three determination methods and the selection of a method for determining the effective swath of the plant protection UAV in mountain orchards suitable for use with the mist nozzle. The three methods for determining the effective swath are as follows:

(1) The evaluation method of droplet density: In agricultural spraying operations carried out by aircraft, the spacing between two points where the target of the operation achieves a droplet deposition density of 15 droplets/cm^2^ or more is the effective swath width (Li et al., 2018).
(2) The evaluation method of 50% effective deposition: According to ASABE standard S341.3 (ASABE S341.3, 2004), the distance between two points where the deposition is 1/2 of the maximum deposition is defined as the effective swath width.
(3) Bilateral 1% coverage evaluation method: The sampling site on either side of the aircraft’s route where the furthest coverage can reach 1% is used as the starting and ending point of the effective swath, and the distance between these two points is the effective swath width.

### 2.6 Statistical data analysis

All statistical analyses were performed using Microsoft Excel 2019 (Microsoft, Redmond, Washington, USA), SPSS26.0 (IBM Corp., Armonk, USA), and Origin2018 (ORIGIN Lab, Northampton, MA, USA) software.

## 3 Results and discussion

### 3.1 UAV operational performance

#### 3.1.1 The penetration coefficient of the canopy

**Table 4** shows the canopy penetration in the vertical direction in the USW and the horizontal direction in the RSW. When the UAV operated along the unidirectional route, the coverage rate on the ground sampling sites, as well as the canopy sampling sites, was relatively high when the flight height was 3 m under the treetop flight mode, at which time the droplet penetration effect was the best, while the penetration coefficient was more uniform at approximately 50% under other operating conditions. The droplet penetration coefficient was relatively high overall, indicating that the droplet spray quality was very good, and it was also more stable at different flight heights and worked better.

**Table 4:** Droplet penetration coefficients on fruit trees under different operating methods.

Mode of operation	Flight mode	Flight parameters	The penetration coefficient/%
Unidirectional	Flying along the tree tops	2m 3m 4m	49 73 51
	Flying along the inter-row	2m 3m 4m	51 49 50
Reciprocating	Flying along the tree tops	20 µm 30 µm 40 µm	76 78 73
	Flying along the inter-row	20 µm 30 µm 40 µm	100 200 74

The flight parameters in the unidirectional operation mode are the flight height, and those in the reciprocating operation mode are the nozzle atomization performance. The canopy penetration was obtained in the vertical direction for the USW and the horizontal direction for the RSW.

When the UAV performed the reciprocating operation, the penetration coefficient was approximately 75%, indicating that there were more droplets on the inner ring of the middle canopy position, and the best penetration coefficient was achieved with a nozzle atomization performance of 30 µm. Under the inter-row flying conditions, the droplet size levels had unusually high penetration coefficient values at 20 µm and 30 µm, reaching 100% and 200%, respectively. Because small droplets are prone to drift, the UAV was flanked by the sampling trees as it flew inter-row, and smaller droplet sizes were more likely to drift to the inner ring of the middle canopy. Smaller droplets were more susceptible to convolution and penetration by the downwash airflow than larger droplets (Wang et al., 2022). As a result, they had more opportunity to deposit in the inner rim of the blade, resulting in a relatively high penetration coefficient.

#### 3.1.2 Distribution span of droplet size

**Figure 6:**
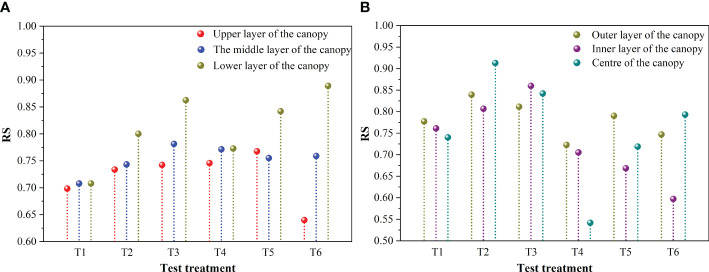
The distribution span of droplet size on fruit trees: **(A)** the distribution span of droplet size at canopy sampling sites in the vertical direction during the unidirectional operation mode, **(B)** the distribution span of droplet size at canopy sampling sites in the horizontal direction during the reciprocating operation mode.

**Figure 6** shows the distribution span of droplet size on canopy sampling sites in a mountain orchard when operating along the unidirectional route versus the reciprocating route. **Figure 6A** shows that the RS was larger in the lower canopy and smaller in the upper canopy when the UAV operated in one direction and that the different canopy positions in the vertical direction had a significant effect on the RS (p=0.017). This may be because the droplets were blocked by the leaves during the settling process, making the uniformity of the droplets near the lower canopy position worse. At the same time, the RS in the lower canopy was highest at a flight height of 4 m compared to flight heights of 2 m and 3 m, at 0.86 (T3) and 0.89 (T6), respectively. In addition, the study also found that with increasing flight height, RS in the upper and middle layers of the canopy increased slowly when flying along the treetops, while RS in the upper and middle canopy locations ranged from 0.64 to 0.77 with no apparent regularity under the inter-row flying, and RS in the lower canopy locations increased significantly under both flight modes. This indicates that flight height has some effect on the distribution span of droplet size in the lower canopy. For the reciprocating operation (**Figure 6B**), the effect of different canopy positions in the horizontal direction on RS was nonsignificant (P=0.686). The analysis revealed that for both treetop flying and inter-row flying, there was a tendency for the RS to increase at the same sampling position as the nozzle atomization performance increased; however, the overall trend was not very clear. This suggests that the nozzle atomization performance also had an effect on the distribution span of droplet size, but the effect on RS was likely to be smaller than that of the flight height.

**Figure 7:**
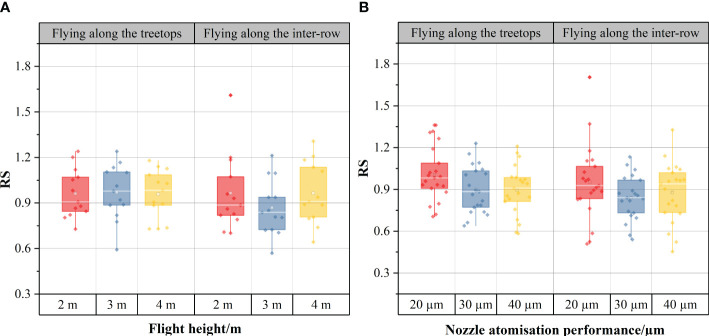
The distribution span of droplet size on ground sampling sites: **(A)** the distribution span of droplet size at different flight heights in the unidirectional operation mode, **(B)** the distribution span of droplet size for different nozzle atomization performances in the reciprocating operation mode.

The uniformity of the droplet distribution on the ground sampling sites when the UAV was operating along the unidirectional and reciprocating routes in the mountain orchard is shown in **Figure 7**. The RS at different flight heights was 0.96 (2 m), 0.97 (3 m), and 0.96 (4 m) when the UAV was operating on the unidirectional route (**Figure 7A**) flying along the treetops, with a relatively close uniformity of droplet distribution. The RS was 0.97 (2 m), 0.87 (3 m), and 0.97 (4 m) during inter-row flying, with the smallest at the flight height of 3 m. When the UAV was operating on the reciprocating route (**Figure 7B**), the RS flying along the treetops versus inter-row flying was 1 (20 µm), 0.9 (30 µm), 0.9 (40 µm), 0.95 (20 µm), 0.84 (30 µm), and 0.88 (40 µm), respectively, and the RS was minimal at an atomization level of 30 µm during inter-row flying. In addition, it was found that the RS measured at the ground sampling sites were relatively close to each other in both unidirectional and reciprocating operations, indicating that the droplet distribution had a more consistent homogeneity. Combining **Figures 6A** and **6B**, it was found that the RS on the ground sampling sites were all greater than 0.8, generally higher than the upper and middle canopy sampling sites, and closer to the RS on the lower canopy sampling sites, indicating that the different canopy positions can have some influence on the uniformity of droplets due to leaf shading.

### 3.2 Selection of the effective swath determination method

The evaluation method of droplet density and the evaluation method of 50% effective deposition are the two most common tests used to determine the effective swath of UAVs. In this study, the first sampling tape treated with different heights was selected for the unidirectional flat test, and the first sampling tape was selected for each of the three flight heights under different flight modes in the unidirectional mountain test to measure the effective swath. The effective swath of the EA-30X UAV was evaluated based on the density of droplet deposition results (**Table 5**). The effective swath distribution range of each sampling tape for each treatment was as follows: ≥11 m (T1–1), ≥11 m (T2–1), ≥11 m (T3–1), ≥11 m (T4–1), ≥10 m (T5–1), ≥10 m (T6–1), ≥11 m (T7–1), ≥12 m (T8–1), and ≥9 m (T9–1). The effective swath of the EA-30X UAV was evaluated based on the droplet deposition results (**Table 6**), and the effective swath distribution range of each sampling tape for each treatment was as follows: 1 to 2 m (T1–1), 1 to 2 m (T2–1), 2 to 3 m (T3–1), 0 to 1 m (T4–1), 2 to 3 m (T5–1), 3 to 4 m (T6–1), 1 to 2 m (T7–1), 0 to 1 m (T8–1) and 2 to 3 m (T9–1).

**Table 5:** The effective swath distribution range for the evaluation method of droplet density.

Sampling location	Sampling tapes for each of the experimental treatments								
	T1–1	T2–1	T3–1	T4–1	T5–1	T6–1	T7–1	T8–1	T9–1
-6m	**17.8**	**55.9**	**62.2**	12.5	13.9	1.5	12	**77.3**	9.8
-5m	**52.9**	**32.2**	**143.2**	**20.1**	8.5	9.4	**49.6**	**17.2**	10.7
-4m	**142**	**65.7**	**202.6**	**53.7**	**28.6**	**73.5**	**97.5**	**16.2**	9.3
-3m	**156.9**	**101.3**	**282.9**	**89.9**	**81.4**	**75.2**	**38.7**	**15.8**	**43.9**
-2m	**246.5**	**275.6**	**264.1**	**64.8**	**66.7**	**205.6**	**27.7**	**90**	**39.6**
-1m	**416.3**	**472.5**	**337.6**	**296**	**128.3**	**176**	**499.6**	**416**	**501.2**
0m	**368.7**	**369.8**	**297.1**	**410.9**	**501.1**	**428.8**	**483.1**	**397.9**	**484.1**
1m	**314.8**	**232.3**	**178.9**	**484.1**	**496.8**	**420.3**	**505.9**	**414.1**	**463.7**
2m	**56.6**	**60.5**	**86.3**	**371.2**	**471.6**	**426**	**116.6**	**202.6**	**244.8**
3m	**83.5**	**18.2**	**37.4**	**157.9**	**370.1**	**389.8**	**149.2**	**38.8**	**245.1**
4m	**26.7**	**19.6**	**34.4**	**48**	**395.6**	**175.5**	**256.8**	**44.9**	**350.9**
5m	**24.4**	**18.5**	**22.4**	**33.3**	**262.9**	**67.3**	**205.9**	**98.1**	**196.4**
6m	1.2	3.4	1.8	**24.2**	**120.9**	**250.9**	**94.4**	**93**	**187.2**

TM-N in the table indicates the Nth sampling tape of the Mth test treatment, and bold numbers indicate droplet deposition densities greater than 15 droplets/cm^2^, i.e., the droplet deposition density that meets the effective swath of the evaluation method of droplet density, in droplets/cm^2^.

**Table 6:** The effective swath distribution range for the evaluation method of 50% effective deposition.

Sampling location	Sampling tapes for each of the experimental treatments								
	T1–1	T2–1	T3–1	T4–1	T5–1	T6–1	T7–1	T8–1	T9–1
-6m	0.008	0.025	0.031	0.008	0.006	0.002	0.004	0.001	0.003
-5m	0.025	0.018	0.092	0.009	0.005	0.006	0.016	0.002	0.004
-4m	0.087	0.046	0.153	0.042	0.008	0.039	0.035	0.004	0.005
-3m	0.088	0.074	0.225	0.027	0.034	0.056	0.02	0.012	0.014
-2m	0.343	0.227	**0.251**	0.027	0.032	0.11	0.021	0.016	0.017
-1m	**0.736**	**0.802**	**0.455**	0.236	0.088	0.132	**1.161**	0.105	**1.087**
0m	**0.485**	**0.719**	**0.343**	0.436	**1.007**	**1.101**	**1.317**	**1.223**	**1.119**
1m	0.272	0.191	0.147	**0.938**	**1.135**	**1.014**	0.478	0.145	**0.871**
2m	0.024	0.027	0.07	0.333	**0.897**	**0.844**	0.059	0.127	0.183
3m	0.041	0.008	0.026	0.103	0.335	**0.708**	0.068	0.152	0.184
4m	0.01	0.009	0.018	0.016	0.367	0.155	0.11	0.129	0.289
5m	0.012	0.009	0.012	0.01	0.145	0.041	0.104	0.045	0.107
6m	0	0.001	0.001	0.01	0.078	0.223	0.034	0.04	0.101

The bolded numbers in the table indicate the droplet deposition that meets the effective swath range of the evaluation method of 50% effective deposition in µL/cm^2^.

There were differences in the results of the effective swath width assessed by the different assessment methods in the same treatment. The results of the effective swath width assessed by the evaluation method of droplet density fluctuated from ≥9 m to ≥12 m, with a large swath width range, while the results of the effective swath width assessed by the evaluation method of 50% effective deposition varied between 0 to 1 m and 3 to 4 m, with a small swath width range, showing a large difference in the swath width results between the two methods. This phenomenon may be explained by the fact that the EA-30X UAV used in the tests used a double-peaked mist nozzle with extremely small droplet sizes, with droplets under 100 µm accounting for approximately 70% of the droplets and only approximately 5% of the droplets above 200 µm (see Section 3.4 for details); therefore, the droplet deposition density on the water-sensitive papers was basically greater than 15 droplets/cm^2^ or more, and the amount of droplet deposition was also relatively small (0.001 to 1.7 µL/cm^2^). Therefore, using these two methods, it was difficult to assess the effective swath of the EA-30X EAVISION plant protection UAV in mountain orchards.

According to the droplet coverage results obtained by the experiment, the effective width of the EA-30X UAV was innovatively evaluated using the bilateral 1% coverage evaluation method, as shown in **Table 7**. The effective swath distribution of each sampling tape for each treatment ranged from 5–6 m (T1–1), 6–7 m (T2–1), ≥8 m (T3–1), 7–8 m (T4–1), ≥9 m (T5–1), ≥10 m (T6–1), ≥7 m (T7–1), ≥7 m (T8–1) and ≥7 m (T9–1). Comparing the results of the three determination methods (**Table 8**), the bilateral 1% coverage evaluation method is close to the effective swath range (7~8 m) of the EA-30X plant protection UAV adapted to the mist nozzle. Furthermore, since the test was carried out in mountainous terrain and the UAV was fitted with a double-peaked mist nozzle (small droplet size), using droplet coverage (the ratio of the target surface area covered by droplets to the total target surface area) for this assessment based on actual operational conditions and operational experience is more accurate. Therefore, in this study, the effective swath was assessed using the bilateral 1% coverage evaluation method on both sides.

**Table 7:** The effective swath distribution range for the bilateral 1% coverage evaluation method.

Sampling location	Sampling tapes for each of the experimental treatments								
	T1–1	T2–1	T3–1	T4–1	T5–1	T6–1	T7–1	T8–1	T9–1
-6m	0.33	1	**1.19**	0.27	0.21	0.06	0.17	0.07	0.14
-5m	0.98	0.65	**3.24**	0.33	0.19	0.2	0.69	0.07	0.16
-4m	**3.09**	**1.59**	**4.98**	**1.25**	0.37	**1.49**	1.46	0.17	0.17
-3m	**3.18**	**2.52**	**7.27**	**1.21**	**1.31**	**1.86**	0.77	0.49	0.61
-2m	**9.21**	**7.27**	**7.57**	**1.06**	**1.25**	**3.97**	0.64	0.64	0.68
-1m	**17.79**	**19.29**	**12.01**	**7.64**	**3.02**	**4.38**	**25.44**	**3.61**	**24.47**
0m	**12.94**	**16.18**	**9.64**	**12.52**	**23.05**	**23.85**	**27.67**	**25.67**	**24.9**
1m	**8.19**	**6.2**	**4.61**	**21.78**	**25.08**	**22.7**	**14.31**	**5.08**	**20.68**
2m	0.97	**1.09**	**2.21**	**10.28**	**21**	**19.99**	**2.13**	**4.4**	**5.87**
3m	1.56	0.3	0.91	**3.55**	**10.3**	**17.59**	**2.7**	**5.42**	**5.83**
4m	0.43	0.37	0.69	0.67	**11.16**	**4.84**	**4.29**	**4.84**	**9.23**
5m	0.47	0.36	0.45	0.46	**5.22**	**1.47**	**3.9**	**1.79**	**3.89**
6m	0.02	0.05	0.04	0.42	**2.75**	**6.97**	**1.41**	**1.74**	**3.74**

The bolded numbers in the table indicate the coverage of the effective swath range that meets the bilateral 1% coverage evaluation.

**Table 8:** Comparison of the results of the three effective swath determination methods.

Test treatment	Results of effective width determination		
	Method for determining the density of droplet deposition	50% effective deposition determination method	Bilateral 1% coverage determination method
T1–1	≥11m	1~2m	5~6m
T2–1	≥11m	1~2m	6~7m
T3–1	≥11m	2~3m	≥8m
T4–1	≥11m	0~1m	7~8m
T5–1	≥10m	2~3m	≥9m
T6–1	≥10m	3~4m	≥10m
T7–1	≥11m	1~2m	≥7m
T8–1	≥12m	0~1m	≥7m
T9–1	≥9m	2~3m	≥7m

The sampling spacing is 1 m.

### 3.3 Effective swath in flatland and mountain scenes

**Figure 8:**
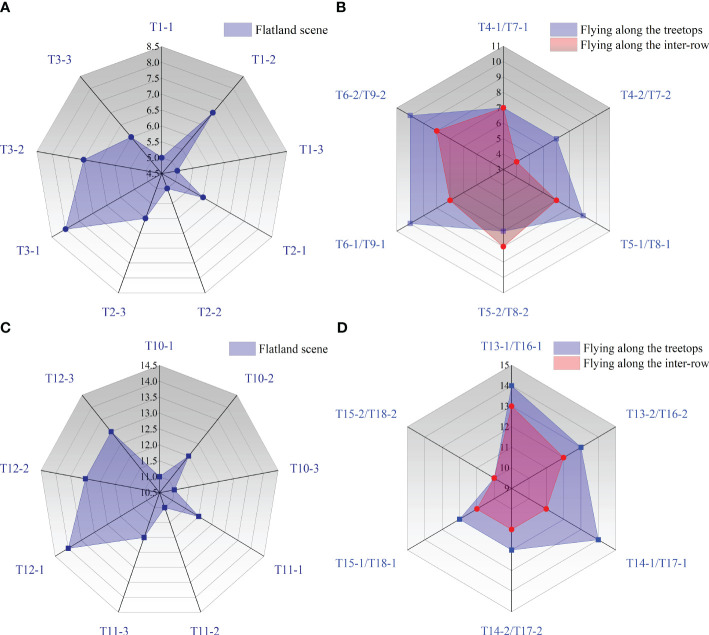
The effective swath width in different scenes: **(A)** the unidirectional effective swath width measured in the flatland scene during unidirectional operation, **(B)** the unidirectional effective swath width measured in the mountain scene during unidirectional operation, **(C)** the reciprocating effective swath width measured in the flatland scene during reciprocating operation, and **(D)** the reciprocating effective swath width measured in the mountain scene during reciprocating operation.

**Figure 8** shows the distribution of the effective swath for the flatland and mountain scenes. The measurement results in the unidirectional operation mode and reciprocating operation mode were the effective swath widths for unidirectional and reciprocating operations, respectively. By clarifying the effective swath range of the UAV in flatland scenarios, it serves as a reference for the experimental design of mountain orchards. As shown in **Figure 8A**, for unidirectional operation at flight heights of 2 m, 3 m, and 4 m, the average unidirectional effective swath widths of the flatland scene were 5.67 m, 5.67 m, and 7 m, the average unidirectional effective swath widths of the mountain scene flight mode for treetop flying were 7 m, 8 m, and 10 m (**Figure 8B**), and the average unidirectional effective swath widths of the flight mode for inter-row flying were 5.5 m, 7.5 m, and 7.5 m, respectively. A comparative analysis revealed that the unidirectional effective swath width for the same flight height for both flight modes in the mountain scene was basically greater than that in the flatland scene, showing that the mountainous slope can affect the unidirectional effective swath width, given the same flight height.

For the reciprocating operation, the average reciprocating effective swath width of the UAV nozzle droplet size in **Figure 8C** increased from 20 µm to 40 µm, the average reciprocating effective swath widths of the flatland scene changed from 11.33 m to 11.67 m and finally increased to 13.33 m, the average reciprocating effective swath widths of the mountain scene flight mode for treetop flying were 13.5 m, 13 m, and 11 m, respectively, and the average reciprocating effective swath width of flight mode for inter-row flying was 12.5 m, 11 m, and 10.5 m. The results showed that the reciprocating effective swath width increased with increasing nozzle droplet size in the flatland scene, probably due to the reciprocating flight of the UAV, with overlapping spray droplets under the two routes, and the higher coverage of the larger droplet size and therefore the increased reciprocating effective swath width. However, the reciprocating effective swath tended to decrease with increasing nozzle droplet size in both flight modes in the mountain scene (**Figure 8D**), indicating that the reciprocating effective swath width was influenced by the slope of the mountain scene. At the same time, for the same nozzle droplet size, as the droplet size increased in the flatland scene, the reciprocating effective swath width increased, but the reciprocating effective swath width in the mountain scene decreased as the droplet size increased, and the trend of the effective swath width change in the two scenes was not the same. It is not possible to analyse the difference between the reciprocating effective swath width in the flatland scene and the mountain scene under the same droplet size conditions. Future experiments will be conducted to address this limitation. In the meantime, the effect of mountain slope size on the effective swath width will continue to be investigated.

### 3.4 Effect of different factors on the effective swath

The effect of the following factors on the effective swath width was based on the analysis of both flight modes with treetop flying and inter-row flying. The USW measured the unidirectional effective swath width at different flight heights, and the RSW measured the reciprocating effective swath width in the direction at different nozzle atomization performances.

#### 3.4.1 Flight height

**Figure 9:**
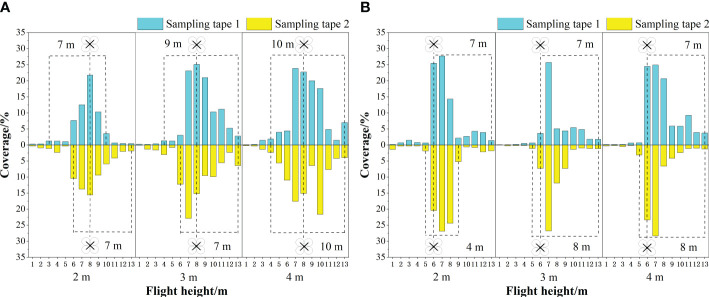
The unidirectional effective swath width at different flight heights for unidirectional operation: **(A)** measured in the flight mode of treetop flying, **(B)** measured in the flight mode of inter-row flying. The numbers 1–13 in the horizontal coordinates indicate the sampling positions at different flight heights.

**Figure 9** shows the results of the effect of different flight heights on the effective swath width for both treetop flying and inter-row flying. The graph shows that the effective spray swath width decreased along both sides of the route. At the same time, the unidirectional effective swath width increased as the UAV flight height increased under the plant protection UAV operating mode along the treetops. With flight heights of 2 m, 3 m, and 4 m, the unidirectional effective swath widths of sampling tape 1 were 7 m, 9 m, and 10 m, respectively, and the unidirectional effective swath widths of sampling tape 2 were 7 m, 7 m, and 10 m, respectively. The unidirectional effective swath width of sampling tape 1 was 7 m, and the unidirectional effective swath width of sampling tape 2 was 4 m, 8 m, and 8 m when the flight height changed from 2 m to 4 m via 3 m under the plant protection UAV flying inter-row.

When the flight height changed from 2 m to 4 m via 3 m, the average value of the unidirectional effective swath width of sampling tape 1 and sampling tape 2 was taken, and the average value of the unidirectional effective swath width was 7 m, 8 m and 10 m when the UAV flew along the treetops and 5.5 m, 7.5 m and 7.5 m when the UAV flew inter-row. Regardless of the kind of flight mode, the average unidirectional effective swath width generally increased with increasing flight height, which was consistent with the actual operational situation. This may be due to the increase in flight height, but when the same nozzle was used, the spray amplitude did not change, and the droplets were displaced more horizontally; therefore, the spraying area became larger. This view is consistent with Zhang et al. (2021). Further analysis reveals that the unidirectional effective swath width measured by the UAV during treetop flying was generally greater than that of the UAV during inter-row flying for the same flight height. The analysis may be because with treetop flying, the droplets from the UAV fell directly on the leaves, while with inter-row flying, the adjacent fruit tree leaves partially blocked the droplets, reducing the range of the unidirectional effective swath.

#### 3.4.2 Nozzle atomization performance

**Figure 10:**
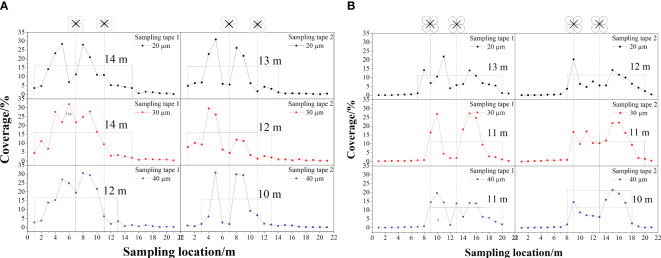
The reciprocating effective swath for different nozzle atomization performances in the reciprocating operation: **(A)** measured in the flight mode of treetop flying, **(B)** measured in the flight mode of inter-row flying.

**Figure 10** shows the results of the effect of different nozzle atomization performances on the effective swath width for both treetop flying and inter-row flying flight modes during RSW. As the test was the reciprocating swath width determination, the reciprocating effective swath width was greater than the unidirectional effective swath width of the USW. The nozzles of the plant protection UAV had nozzle droplet sizes of 20 µm, 30 µm, and 40 µm, with reciprocating effective swath widths of 14 m (20 µm), 14 m (30 µm), and 12 m (40 µm) for sampling tape 1 and 13 m (20 µm), 12 m (30 µm) and 10 m (40 µm) for sampling tape 2, respectively, in the treetop flight mode. Under the inter-row flight mode of the plant protection UAV, the reciprocating effective swath widths of sampling tape 1 were 13 m (20 µm), 11 m (30 µm) and 11 m (40 µm), and the reciprocating effective swath widths of sampling tape 2 were 12 m (20 µm), 11 m (30 µm) and 10 m (40 µm), respectively. The analysis reveals that the reciprocating effective swath width decreased with increasing nozzle droplet size in both flight modes. The increased droplet size and increased droplet weight reduce droplet drift, increasing the number of droplets falling below the UAV and therefore reducing the reciprocating effective swath width. A previous study (Yao et al., 2021) using helicopter operations found that the effective swath width varied with the nozzle orifice size, suggesting that the nozzle atomization performance can affect the effective swath width.

In addition, under the same nozzle droplet size conditions, the mean value of the reciprocating effective swath widths measured by the UAV during treetop flying (13.5 m, 13 m, 11 m) was larger than that measured by the UAV during inter-row flying (12.5 m, 11 m, 10.5 m), which was the same regulation as the unidirectional effective swath rule obtained for different flight modes at the same height in the 3.2.1 unidirectional swath width determination above. The route spacing was 4 m for the reciprocating operation, but the results of the data showed that the intermediate route coverage values were all greater than 1%. This indicated that the route spacing can continue to be increased to ensure that the best swath width is obtained when the same agent is sprayed. The exact spacing of the additional routes will need further experimental study in the future to make full use of the sprayed pesticides and reduce environmental pollution.

### 3.5 Droplet-size spectrum and position of the effective swath

**Figure 11:**
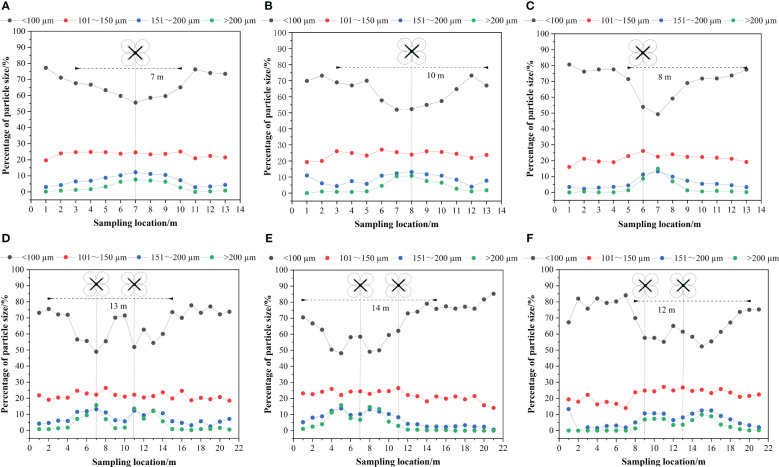
Droplet size distribution and the effective swath width distribution: **(A)** unidirectional operation in the flatland scene, **(B)** unidirectional operation for treetop flight in the mountain scene, **(C)** unidirectional operation for inter-row flight in the mountain scene, **(D)** reciprocating operation in the flatland scene, **(E)** reciprocating operation for treetop flight in the mountain scene, and **(F)** reciprocating operation for inter-row flight in the mountain scene. The effective swath width is the average value for the same flight mode.

The percentage of droplet size distributions for the flatland and mountain scenes are shown in **Figure 11** for both the unidirectional and reciprocating routes. The analysis shows that the percentage of the droplet size distribution decreased as the droplet size level increased for both flatland and mountain scenes. At the same time, the proportion of droplet sizes smaller than 100 µm on the ground sampling sites below or near the UAV rotor was approximately 50%, which was lower than that on both sides of the route (approximately 80%), and the overall trend decreased and then increased with the change in the UAV route position, but the proportion of droplet sizes larger than 200 µm on the ground sampling sites was the opposite, and the overall trend increased and then decreased with the change in the UAV route position. This phenomenon was consistent with the findings of Ahmad et al. (2020), who found that droplet diameter decreased from the centerline of flight and showed a slight tendency to fluctuate at the centerline. In addition, as in the case of unidirectional operation, the percentage of droplet sizes smaller than 100 µm at sampling locations below or near the route was smaller than that at other sampling locations when the UAV was reciprocating. The reason for this analysis is that the sampling sites below the UAV route were more influenced by the downwash airflow of the rotor, and the smaller droplet sizes were easier to drift, while the larger droplet sizes were less likely to be affected by the rotor airfield and roll up, so the distribution ratio of droplets with a high droplet size level below the UAV was increased compared to the sides of the route.

In addition, this regulation is more obvious in the mountain scene, in which the highest values of droplet size larger than 200 µm were 7.66%, 10.75%, and 14.96% in the flatland scene and mountain scene for treetop and inter-row flights, respectively, while the smallest values of droplet size less than 100 µm decreased from 55.61% to 51.91% to 49.30%, respectively. The results show that the rotor airflow of UAVs in mountain scenes has a greater influence on the droplet size than that in flatland scenes, and inter-row flight in mountain scenes is more obvious than treetop flight. Some scholars have addressed the effect of droplet deposition on both treetop and inter-row flight modes. Meng et al. (2020) measured the effect of droplet deposition under treetop and inter-row flight modes based on two different tree shapes and found that different tree shapes had different droplet coverage under different flight modes. Qi et al. (2022) established a precision targeting device, BUAV (treetop flight), compared to a conventional multirotor UAV, CUAV (inter-row flight), but there was a lack of contrast between the different flight modes. The effect of different flight modes on the effective swath was analysed, and it can be found that when the UAV flew inter-row, whether it was unidirectional operation or reciprocating operation, the position of the effective swath was not uniform on either side of the UAV’s route but was approximately 3 m to 4 m off the UAV’s course in the downhill direction. The reason for this phenomenon is that when the UAV flew inter-row, unlike when it flew along the tops of the trees (Dou et al., 2023), some of the droplets sprayed by the UAV landed directly on the leaves of the fruit trees below the UAV, and the low planting density of the mountain orchards, influenced by the rotor airflow of the UAV (Zhang et al., 2023) and the natural wind on the mountain, led to a shift in the effective swath along the downhill direction. Therefore, to avoid the misspraying of fruit trees in the direction of the hill, the heavy spraying of fruit trees in the downhill direction, and spraying agents falling on nontarget crops, causing crop damage and environmental pollution, it is recommended to fly along the treetops when operating in the Nanguo pear orchard mountain settings.

## 4 Conclusions

In this study, the EA-30X UAV was used to select a suitable determination method for mist nozzles by comparing three commonly used methods for determining effective swaths. The operating parameters, such as flight height, nozzle atomization level, and flight mode, were varied to investigate the effects on the effective swath width in mountain orchards and to preliminarily analyse the differences in the effective swath width between flatland and mountain settings with the same operating parameters. This study was based on an EAVISION UAV and mountain orchard scenes, which were targeted and may not be applicable to other scenes; further research will be needed in the future. From the test results, the following conclusions can be drawn:

(1) In the unidirectional operation, the UAV flew along the treetops, with the best penetration of 0.73 at a flight height of 3 m. For the distribution span, the RS was less in the upper canopy than in the lower canopy and was greater at a flight height of 4 m than at the other two flight heights (2 m and 3 m). The RS on the ground sampling site was the smallest during inter-row flying and the flight height was 3 m, and the nozzle atomization performance was 30 µm with the best penetration effect when the UAV reciprocated the operation, which was 0.78 and 2 for treetop flying and inter-row flying, respectively. At the same time, the RS at the position of the outer ring in the middle of the canopy was generally higher than that in the inner canopy. In addition, the RS at the canopy sampling sites was smaller than that at the ground sampling sites. The results showed that different canopy positions had a greater effect on the uniformity of droplet distribution. However, overall, the penetration coefficient was higher, the RS data fluctuated less when the operating parameters were the same, and the UAV operating performance was more stable.
(2) The bilateral 1% coverage evaluation method applies to the double-peaked mist nozzle or nozzles with smaller droplet sizes for effective swath determination. The effective swath was influenced by the mountainous slope, with the effective swath width of the unidirectional spray applied in both flight modes being greater in the mountain scene (7 to 10 m for treetop flying and 5.5 to 7.5 m for inter-row flying) than in the flatland scene (5 to 7 m) at the same flight height. The results showed that the mountain slope had a certain influence on the effective swath, but the specific change trend requires further study in the future. In the mountain scene where the UAV operated on the unidirectional route, the unidirectional effective swath width for treetop and inter-row flight both increased with flight height. When operating along the reciprocating route, the atomization level increased and the reciprocating effective swath width decreased. In addition, regardless of whether the UAV was operating in unidirectional or reciprocating, the effective swath under the flight mode along the treetops (7–10 m and 11–13.5 m for unidirectional and reciprocating operations, respectively) was greater than that along the inter-row (5.5–7.5 m and 10.5–12.5 m for unidirectional and reciprocating operations, respectively), which indicated that the flight modes (treetop and inter-row flight) had the effective swath were significantly affected.
(3) The percentage of small droplets below the UAV’s route was lower than that on either side of the route, and the percentage of large droplet particles was higher. When the EA-30X UAV operated under inter-row flight, the position of the effective swath was not symmetrically distributed to the left and right of the UAV’s course but approximately 3 m to 4 m to the side of the UAV’s course downhill. Therefore, to obtain better operational results, it is recommended that the UAV be operated in mountain orchards while flying along treetops to ensure that the best effective swath width is obtained when applying the same agent and to improve the application effect of the UAV.

## Data availability statement

The raw data supporting the conclusions of this article will be made available by the authors, without undue reservation.

## Author contributions

YL: Conceptualization, Methodology, Software, Validation, Visualization, Writing – original draft. WY: Funding acquisition, Investigation, Project administration, Supervision, Writing – review & editing. SG: Investigation, Writing – review & editing. HY: Writing – review & editing. ZY: Writing – review & editing. SM: Writing – review & editing. DC: Resources, Supervision, Writing – review & editing. CC: Funding acquisition, Supervision, Writing – review & editing.

## References

[B1] Ahmad F. Qiu B. J. Dong X. Y. Ma J. Huang X. Ahmed S.. (2020). Effect of operational parameters of UAV sprayer on spray deposition pattern in target and off-target zones during outer field weed control application. Comput. Electron Agric. 172, 10. doi: 10.1016/j.compag.2020.105350

[B2] ASABE S341.3. (2004). FEB04-Procedure for measuring distribution uniformity and calibrating granular broadcast spreaders (St. Joseph, MI: American Society of Agricultural and Biological Engineers).

[B3] Biglia A. Grella M. Bloise N. Comba L. Mozzanini E. Sopegno A.. (2022). UAV-spray application in vineyards: Flight modes and spray system adjustment effects on canopy deposit, coverage, and off-target losses. Sci. Total Environ. 845, 17. doi: 10.1016/j.scitotenv.2022.157292 35820523

[B4] Brown C. R. Giles D. K. (2018). Measurement of pesticide drift from unmanned aerial vehicle application to a vineyard. Trans. ASABE 61, 1539–1546. doi: 10.13031/trans.12672

[B5] Chen S. Lan Y. Li J. X X. Wang Z. Peng B. (2017). Evaluation and test of effective spraying width of aerial spraying on plant protection UAV. Trans. Chin. Soc. Agric. Eng. (Transactions CSAE) 33, 82–90. doi: 10.11975/j.issn.1002-6819.2017.07.011

[B6] Chen S. D. Lan Y. B. Zhou Z. Y. Ouyang F. Wang G. B. Huang X. Y.. (2020). Effect of droplet size parameters on droplet deposition and drift of aerial spraying by using plant protection UAV. Agronomy." 10, 15. doi: 10.3390/agronomy10020195

[B7] Chen C. C. Li S. G. Wu X. Y. Wang Y. X. Kang F. (2022). Analysis of droplet size uniformity and selection of spray parameters based on the biological optimum particle size theory. Environ. Res. 204, 8. doi: 10.1016/j.envres.2021.112076 34555405

[B8] Chen P. C. Ouyang F. Wang G. B. Qi H. X. Xu W. C. Yang W. G.. (2021). Droplet distributions in cotton harvest aid applications vary with the interactions among the unmanned aerial vehicle spraying parameters. Ind. Crops Prod. 163, 10. doi: 10.1016/j.indcrop.2021.113324

[B9] Dou Z. Fang Z. Han X. Zeeshan M. Liu Y. Lan Y. (2023). Effects of spray adjuvants and operation modes on droplet deposition and elm aphid control using an unmanned aerial vehicle. Int. J. Agric. Biol. Eng. 16, 1–9. doi: 10.25165/j.ijabe.20231602.7424

[B10] Giles D. Billing R. (2015). Deployment and performance of a UAV for crop spraying. Chem. Eng. Trans. 44, 307–312. doi: 10.3303/CET1544052

[B11] Guo S. Li J. Y. Yao W. X. Hu X. D. Wei X. Long B.. (2021). Optimization of the factors affecting droplet deposition in rice fields by rotary unmanned aerial vehicles (UAVs). Precis. Agric. 22, 1918–1935. doi: 10.1007/s11119-021-09818-7

[B12] Guo S. Yao W. X. Xu T. Y. Ma H. Sun M. J. Chen C. L.. (2022). Assessing the application of spot spray in Nanguo pear orchards: Effect of nozzle type, spray volume rate and adjuvant. Pest Manage. Sci. 78, 3564–3575. doi: 10.1002/ps.6999 35598076

[B13] Hou C. J. Tang Y. Luo S. M. Lin J. T. He Y. Zhuang J. J.. (2019). Optimization of control parameters of droplet density in citrus trees using UAVs and the Taguchi method. Int. J. Agric. Biol. Eng. 12, 1–9. doi: 10.25165/j.ijabe.20191204.4139

[B14] Hussain M. Wang Z. Huang G. M. Mo Y. Kaousar R. Duan L. S.. (2022). Comparison of droplet deposition, 28-homobrassinolide dosage efficacy and working efficiency of the unmanned aerial vehicle and knapsack manual sprayer in the maize field. Agronomy." 12, 16. doi: 10.3390/agronomy12020385

[B15] Itmec M. Bayat A. Bolat A. Toraman M. C. Soysal A. (2022). Assessment of spray drift with various adjuvants in a wind tunnel. Agronomy." 12, 16. doi: 10.3390/agronomy12102377

[B16] Jiang Y. He X. Song J. Liu Y. Wang C. Li T.. (2022). Comprehensive assessment of intelligent unmanned vehicle techniques in pesticide application: A case study in pear orchard. Front. Plant Sci. 13. doi: 10.3389/fpls.2022.959429 PMC9445492 36082299

[B17] Li L. L. Hu Z. H. Liu Q. J. Yi T. C. Han P. Zhang R. R.. (2022). Effect of flight velocity on droplet deposition and drift of combined pesticides sprayed using an unmanned aerial vehicle sprayer in a peach orchard. Front. Plant Sci. 13. doi: 10.3389/fpls.2022.981494 PMC9559834 36247584

[B18] Li L. Liu Y. He X. Song J. Zeng A. Wang Z. C.. (2018)Assessment of spray deposition and losses in the apple orchard from agricultural unmanned aerial vehicle in China. 2018 ASABE Annu. Int. Meeting.doi: 10.13031/aim.201800504

[B19] Lou Z. X. Xin F. Han X. Q. Lan Y. B. Duan T. Z. Fu W. (2018). Effect of unmanned aerial vehicle flight height on droplet distribution, drift and control of cotton aphids and spider mites. Agronomy." 8, 13. doi: 10.3390/agronomy8090187

[B20] Martin D. E. Latheef M. A. (2022). Payload capacities of remotely piloted aerial application systems affect spray pattern and effective swath. Drones." 6, 21. doi: 10.3390/drones6080205

[B21] Martin D. E. Woldt W. E. Latheef M. A. (2019). Effect of application height and ground speed on spray pattern and droplet spectra from remotely piloted aerial application systems. Drones." 3, 21. doi: 10.3390/drones3040083

[B22] Meng Y. H. Su J. Y. Song J. L. Chen W. H. Lan Y. B. (2020). Experimental evaluation of UAV spraying for peach trees of different shapes: Effects of operational parameters on droplet distribution. Comput. Electron Agric. 170, 12. doi: 10.1016/j.compag.2020.105282

[B23] Qi P. He X. K. Liu Y. J. Ma Y. Wu Z. M. Wang J. W. (2022). Design and test of target-oriented profile modeling of unmanned aerial vehicle spraying. Int. J. Agric. Biol. Eng. 15, 85–91doi: doi.org 10.25165/j.ijabe.20221503.6753

[B24] Qi P. Zhang L. T. Wang Z. C. Han H. Mueller J. Li T.. (2023). Effect of operational parameters of unmanned aerial vehicle (UAV) on droplet deposition in trellised pear orchard. Drones." 7, 24. doi: 10.3390/drones7010057

[B25] Qin W. Xue X. Zhang S. Gu W. Wang B. (2018). Droplet deposition and efficiency of fungicides sprayed with small UAV against wheat powdery mildew. Int. J. Agric. Biol. Eng. 11, 27–32. doi: 10.25165/j.ijabe.20181102.3157

[B26] Ran X. Li T. Xu S. Zhong Y. Wang X. Feng Y.. (2022). “Application comparison of three UAVs in the control of pests and diseases of citrus in guangxi,” in International conference on guidance, navigation and control (Springer Nature Singapore, Singapore), 4643–4650. doi: 10.1007/978-981-19-6613-2_450

[B27] Richardson B. Rolando C. A. Kimberley M. O. Strand T. M. (2019). Spray application efficiency from a multi-rotor unmanned aerial vehicle configured for aerial pesticide application. Trans. ASABE 62, 1447–1453. doi: 10.13031/trans.13509 31595645

[B28] Sarri D. Martelloni L. Rimediotti M. Lisci R. Lombardo S. Vieri M. (2019). Testing a multi-rotor unmanned aerial vehicle for spray application in high slope terraced vineyard. J. Agric. Eng. 50, 38–47. doi: 10.4081/jae.2019.853

[B29] Sinha R. Johnson J. Power K. Moodie A. Warhurst E. Barbosa R. (2022). Understanding spray attributes of commercial UAAS as impacted by operational and design parameters. Drones 6, 20. doi: 10.3390/drones6100281

[B30] Souza F. G. Portes M. F. Silva M. V. Teixeira M. M. Furtado Júnior M. R. (2022). Impact of sprayer drone flight height on droplet spectrum in mountainous coffee plantation. Rev. Bras. eng. agríc. ambient. 26, 901–906. doi: 10.1590/1807-1929/agriambi.v26n12p901-906

[B31] Tang Y. Hou C. J. Luo S. M. Lin J. T. Yang Z. Huang W. F. (2018). Effects of operation height and tree shape on droplet deposition in citrus trees using an unmanned aerial vehicle. Comput. Electron Agric. 148, 1–7. doi: 10.1016/j.compag.2018.02.026

[B32] Wang G. B. Han Y. X. Li X. Andaloro J. Chen P. C. Hoffmann W. C.. (2020). Field evaluation of spray drift and environmental impact using an agricultural unmanned aerial vehicle (UAV) sprayer. Sci. Total Environ. 737, 13. doi: 10.1016/j.scitotenv.2020.139793 32526578

[B33] Wang X. N. He X. K. Song J. L. Wang Z. C. Wang C. L. Wang S. L.. (2018). Drift potential of UAV with adjuvants in aerial applications. Int. J. Agric. Biol. Eng. 11, 54–58. doi: 10.25165/j.ijabe.20181105.3185

[B34] Wang C. L. He X. K. Zeng A. J. Andreas H. Supakorn W. Qiao B. Y.. (2020). Measuring method and experiment on spray drift of chemicals applied by UAV sprayer based on an artificial orchard test bench. Trans. Chin. Soc Agric. Eng. 36, 56–66. doi: 10.11975/j.issn.1002-6819.2020.13.007

[B35] Wang C. L. Herbst A. Zeng A. J. Wongsuk S. Qiao B. Y. Qi P.. (2021). Assessment of spray deposition, drift and mass balance from unmanned aerial vehicle sprayer using an artificial vineyard. Sci. Total Environ. 777, 14. doi: 10.1016/j.scitotenv.2021.146181 33689892

[B36] Wang J. Lan Y. B. Zhang H. H. Zhang Y. L. Wen S. Yao W. X.. (2018). Drift and deposition of pesticide applied by UAV on pineapple plants under different meteorological conditions. Int. J. Agric. Biol. Eng. 11, 5–12. doi: 10.25165/j.ijabe.20181106.4038

[B37] Wang C. L. Liu Y. Zhang Z. H. Han L. Li Y. F. Zhang H.. (2022). Spray performance evaluation of a six-rotor unmanned aerial vehicle sprayer for pesticide application using an orchard operation mode in apple orchards. Pest Manage. Sci. 78, 2449–2466. doi: 10.1002/ps.6875 35306733

[B38] Wang J. Ma C. Chen P. Yao W. Yan Y. Zeng T.. (2023). Evaluation of aerial spraying application of multi-rotor unmanned aerial vehicle for Areca catechu protection. Front. Plant Sci. 14. doi: 10.3389/fpls.2023.1093912 PMC10011446 36925752

[B39] Xavier T. W. F. Souto R. N. V. Statella T. Galbieri R. Santos E. S. Suli G. S.. (2019). Identification of ramularia leaf blight cotton disease infection levels by multispectral, multiscale UAV imagery. Drones." 3, 14. doi: 10.3390/drones3020033

[B40] Yao W. X. Guo S. Wang J. Chen C. L. Yu F. H. Li X.. (2022). Droplet deposition and pest control efficacy on pine trees from aerial application. Pest Manage. Sci. 78, 3324–3336. doi: 10.1002/ps.6959 35491531

[B41] Yao W. Guo S. Yu F. Du W. Meng Y. Wang J.. (2021). Droplet deposition and spatial drift distribution characteristics of aerial spraying based on the determination of effective swath. Int. J. Precis Agric. Aviat 4, 36–43. doi: 10.33440/j.ijpaa.20210401.145

[B42] Yao W. X. Lan Y. B. Wen S. Zhang H. H. Zhang Y. L. Wang J.. (2019). Evaluation of droplet deposition and effect of variable-rate application by a manned helicopter with AG-NAV Guia system. Int. J. Agric. Biol. Eng. 12, 172–178. doi: 10.25165/j.ijabe.20191201.4039

[B43] Zhang D. Chen L. Zhang R. Hoffmann W. C. Xu G. Lan Y. B.. (2015). Evaluating effective swath width and droplet distribution of aerial spraying systems on M-18B and Thrush 510G airplanes. Int. J. Agric. Biol. Eng. 8, 21–30. doi: 10.3965/j.ijabe.20150802.1493

[B44] Zhang P. Deng L. Lyu Q. He S. L. Yi S. L. Liu Y. D.. (2016). Effects of citrus tree-shape and spraying height of small unmanned aerial vehicle on droplet distribution. Int. J. Agric. Biol. Eng. 9, 45–52. doi: 10.3965/j.ijabe.20160904.2178

[B45] Zhang P. Wang K. J. Lyu Q. He S. L. Yi S. L. Xie R. J.. (2017). Droplet distribution and control against citrus leafminer with UAV spraying. Int. J. Robot. Autom. 32, 299–307. doi: 10.2316/Journal.206.2017.3.206-4980

[B46] Zhang H. Wen S. Chen C. Liu Q. Xu T. Chen S.. (2023). Downwash airflow field distribution characteristics and their effect on the spray field distribution of the DJI T30 six-rotor plant protection UAV. Int. J. Agric. Biol. Eng. 16, 10–22. doi: 10.25165/j.ijabe.20231602.8094

[B47] Zhang P. Zhang W. Sun H. T. He F. G. Fu H. B. Qi L. Q.. (2021). Effects of spray parameters on the effective spray width of single-rotor drone in sugarcane plant protection. Sugar Tech. 23, 308–315. doi: 10.1007/s12355-020-00890-3

[B48] Zhou H. Yao W. Su D. Guo S. Zheng Z. Yu Z.. (2024). Application of a centrifugal disc fertilizer spreading system for UAVs in rice fields. Heliyon. 10, e29837. doi: 10.1016/j.heliyon.2024.e29837 38681536 PMC11053224

